# *Candida tropicalis* ZD-3 prevents excessive fat deposition by regulating ileal microbiota and bile acids enterohepatic circulation in broilers

**DOI:** 10.3389/fmicb.2024.1419424

**Published:** 2024-08-14

**Authors:** Jiaqi Feng, Fang Wang, Shanshan Nan, Lijing Dou, Xiaotong Pang, Junli Niu, Wenju Zhang, Cunxi Nie

**Affiliations:** ^1^College of Animal Science and Technology, Shihezi University, Shihezi, China; ^2^Animal Husbandry and Veterinary Workstation of the 8th Division, Shihezi, China

**Keywords:** *Candida tropicalis* ZD-3, gut microbiota, bile acid metabolism, lipid metabolism, yellow-feathered broiler

## Abstract

**Introduction:**

Evidence suggests that the dietary intake of *Candida tropicalis*ZD-3 (ZD-3) has various health benefits, but the treatment mechanisms and effects remain unclear. The aim of this study investigates the effect of ZD-3 on reducing fat deposition in broilers and the underlying mechanism.

**Methods:**

180 one-day-old, yellow-feathered broilers were randomly divided into three groups: control (CON) group fed a basal diet, an active *Candida tropicalis* ZD-3 (ZD) group supplemented with ZD, and a heat-inactivated Candida tropicalis ZD-3 (HZD) group supplemented with HZD. The experiment lasted for 28 d.

**Results:**

The ZD and HZD treatments significantly reduced the abdominal fat index (*p* < 0.05), decreased TG levels in serum and liver (*p* < 0.05), altered the ileal microbial composition by reducing the Firmicutes to Bacteroidetes (F/B) ratio. Additionally, the ZD and HZD treatments reduced liver cholesterol by decreasing ileal FXR-FGF19 signaling and increasing liver FXR-SHP signaling (*p* < 0.05). The ZD and HZD treatments also changed liver PC and TG classes lipid composition, regulating liver lipid metabolism by promoting TG degradation and modulating the signal transduction of the cell membrane.

**Discussion:**

Overall, ZD-3 was effective in improving lipid metabolism in broilers by regulating the ileal microbial composition and BAs enterohepatic circulation. This study provides a theoretical basis for the development and application of ZD-3 for the regulation of lipid metabolism in broilers.

## 1 Introduction

In the poultry industry, high energy diets and genetic breeding methods are used to artificially select chickens to achieve unexampled feed conversion ratios and growth rates (Tallentire et al., 2016). Fast-growing broilers are frequently linked to excessive abdominal fat deposition because one unit of fat consumed for deposition provides three times as much energy as one unit of lean meat consumed for deposition, a trait detrimental to the interests of producers and consumers (Nie et al., 2015). In broilers, more than 85% of abdominal fat is considered unnecessary because it is viewed as a waste of dietary energy (Fouad and El-Senousey, 2014). Excessive fat deposition can lead to a fatty liver, which is associated with high mortality and morbidity in broilers. Global broiler production yields approximately 3 million tons of abdominal fat per year, leading to many economic losses in the poultry industry and posing a significant challenge to sustainable agricultural profitability (Wen et al., 2019). Researchers have discovered that abdominal fat cells exhibit greater metabolic activity and possess higher heritability (0.82) than breast and leg muscles, resulting in fat accumulation (Abdalla et al., 2018). Accumulation of excess fat has become a perplexing and increasingly prevalent problem. Consequently, understanding the underlying mechanisms that contribute to excessive fat deposition has garnered significant attention.

When consumed in adequate amounts, probiotics offer health benefits to the host and have preclinical potential for reducing obesity (Larsen et al., 2023). Therefore, probiotics are considered a non-invasive but potentially effective treatment for obesity and related metabolic diseases (Delzenne et al., 2011). Recently, the cholesterol reducing effects of probiotics have garnered significant research interest, and consumers prefer their safety, high efficacy, and cost-effectiveness to pharmaceutical interventions (Tang et al., 2021). Many probiotic formulations have been developed to control cholesterol levels. However, most studies have used live bacteria. Additionally, studies have suggested that postbiotics also play a role (Salminen et al., 2021). The term postbiotics is a newly recognized concept encompassing various bioactive substances, such as inactive or heat-inactivated microbial cells, cellular components, and metabolic byproducts, that have the potential to promote beneficial biological effects when ingested by consumers (Cuevas-González et al., 2020). Research on postbiotics is still in its early stages, but they have been found to have a significant impact on regulating fat metabolism (Depommier et al., 2019).

In our previous study, we found that diets fermented with ZD-3 were effective in reducing total cholesterol (TC) levels and had the potential to modulate abdominal fat morphology and lipid metabolism in early white feathered broilers but the effect was not evident in later stages (Niu et al., 2020). However, the mechanism of action and the effect of heat-inactivated ZD-3 on fat metabolism remain unclear. Probiotics modulate lipid metabolism by regulating intestinal microbiota and their associated metabolites. The alteration in the gut microbiota, often referred to as the “second brain,” can affect the energy regulation, glucose (GLU) equilibrium, and lipid metabolism of the host (Li et al., 2020). It has been shown that interactions between host ileal microbiota and bile acid (BA) metabolism ameliorate hyperlipidemia and related metabolic diseases (Wang Y. et al., 2023). The primary factor contributing to hyperlipidemia is the accumulation of lipids in the bloodstream and liver. Therefore, the principal approach for treating hyperlipidemia involves restoring the metabolism of lipids, particularly cholesterol and BAs (Zhang Y. et al., 2022). BAs regulate host metabolism and immunity through the farnesoid X receptor (FXR) activated by BAs. Probiotics reduce BA absorption and promote liver bile acid synthesis through the ileum liver FXR-fibroblast growth factor 19 (FGF19) axis (Pathak et al., 2018). The liver is a crucial organ for energy metabolism and plays a significant role in maintaining the lipid balance by regulating lipid synthesis and breakdown (Feng et al., 2020). Lipidomics allows for the identification and evaluation of how lipids, either independently or in conjunction with proteins, regulate cellular and subcellular functions such as signaling and gene expression. The utilization of lipidomic analysis of the liver has the potential to provide new perspectives for investigating lipid metabolism. Hence, the utilization of probiotics to regulate the gut microbiota and improve liver lipid metabolism is a promising research field.

In this study, we evaluated the regulatory effects of active and heat-inactivated ZD-3 on lipid metabolism and its effects on ileum microbiota and BA metabolism and then studied lipid changes in the liver by using lipidomics, providing a theoretical basis for the application and development of ZD-3 and the reduction of fat deposition in broilers.

## 2 Materials and methods

### 2.1 Treatment of *Candida tropicalis* ZD-3

The strains of ZD-3 were obtained from the College of Animal Science & Technology Shihezi University (Shihezi, China). The activated strains were inoculated with YPD at 37°C for 24 h. The ZD-3 were harvested using centrifugation at 4,000 g for 10 min and then washed twice with phosphate buffered saline (pH = 7.4). Next, *Candida tropicalis* ZD-3 (ZD) was obtained by lyophilization (1 × 10^9^ CFU/kg). Heat-inactivated *Candida tropicalis* ZD-3 (HZD) were prepared from the bacterial pellets by subjecting them to a 90°C water bath for 30 min and then lyophilization. Plate culture analysis confirmed the absence of active bacterial growth.

### 2.2 Animals and experimental design

Animal experiments were conducted in accordance with China’s national guidelines and in compliance with the regulations of the Bioethics Committee of Shihezi University (Xinjiang, China) (Approval no: A2023-202). In total, 180 one-day-old male, yellow-feathered broilers were obtained from a business incubator (Xinjiang Taikun Group Co., Ltd.) and randomly divided into three groups, each consisting of six replicates, with 10 birds in each replicate. Broilers in the CON group were fed a basal diet. Broilers in the ZD group were fed the basal diet with added active *Candida tropicalis* ZD-3 (1 × 10^9^ CFU/kg). Broilers in the HZD group were fed a basal diet containing heat-inactivated *Candida tropicalis* ZD-3 (1 × 10^9^ CFU/kg). The compositions and nutrient levels of the diets are listed in Table 1. The basal diet complied with the nutritional requirements of the yellow-feathered broiler chicks (NY/T 3645–2020). Broilers were raised in multilevel coops. Each coop contained three levels, five cages per level, and five birds in each cage. Each cage was equipped with two nipple drinkers and one feeder. The broilers had *ad libitum* access to water to fulfill their nutritional requirements. The cages were under 24 h lighting, and the temperature was initially set at 35°C, gradually decreasing to 25°C by the fourth week. The experiment lasted for 28 d.

**Table 1:** Diet compositions and nutrient contents of the experimental diets.

Item	1–28 d
**Ingredients, %DM**	
Yellow corn	54.80
Soybean meal	37.70
Vegetable oil	2.50
Premix^a^	5.00
**Nutrient content, %DM**	
Metabolizable energy, MJ/kg	12.37
Crude protein	20.98
Ether extract	5.07
Crude ash	7.12
Crude fiber	3.41
Calcium	1.01
Total phosphorus	0.71
Available phosphate	0.45
Methionine	0.53
Methionine + cysteine	0.95
Threonine	0.86
Lysine	1.31

a Premix provided the following per kg of diets: Ca 8.1 g/kg; P 2.97 g/kg; Nacl 2.90 g/kg; Lys 0.256 g/kg; Met 1.813 g/kg; VA 8800 IU/kg; VD 3000 IU/kg; VE 30 mg/kg; VK_2_ 1.65 mg/kg; VB_1_ 2.5 mg/kg; VB_2_ 6.6 mg/kg; VB_3_ 11 mg/kg; VB_4_ 500 mg/kg; VB_5_ 60 mg/kg; VB_6_ 4.0 mg/kg; VH 0.2 mg/kg; VB_11_ 1.0 mg/kg; VB_12_ 0.02 mg/kg; VC 50 mg/kg; Fe^2+^80.0 mg/kg; Cu^2+^ 8.0 mg/kg; Zn^2+^ 60.0 mg/kg; Mn^2+^ 70.0 mg/kg; I 0.5 mg/kg; Se^4+^ 0.3 mg/kg.

### 2.3 Sample collection

At 28 days, after a 12 h fast, six broilers were randomly selected from each treatment group (one bird from each replicate) and sacrificed using cervical dislocation. The broilers were weighed, and blood samples (obtained from the jugular vein), liver tissue, abdominal adipose tissue, and ileal contents were collected. The blood was centrifuged at 3,000 g for 10 min at 4°C to separate the serum, which was stored at −20°C for future use. The liver and abdominal fat were collected and weighed; a portion of each was fixed in 4% paraformaldehyde, and the remainder was frozen in liquid nitrogen and stored at −80°C. Ileal contents were also collected, frozen in liquid nitrogen, and stored at −80°C for subsequent analysis. Feces were collected and mixed in repeat units 3 days before slaughter.

### 2.4 Growth performance

Body weight and feed intake of broilers were monitored weekly in a replicated fashion. These measurements were used to calculate the average daily feed intake (ADFI), average daily gain (ADG), and feed: gain ratio (F/G).

### 2.5 Carcass trait

Carcass, liver, and abdominal fat were measured and reported as a proportion of the live weight. The thickness of the subcutaneous fat (encompassing the skin) in front of the caudal vertebra was determined using Vernier calipers.

### 2.6 Serum and liver lipid indices and fecal total BAs concentration

TC, triglyceride (TG), high-density lipoprotein cholesterol (HDL-C), low-density lipoprotein cholesterol (LDL-C), and GLU in serum; TC, TG, HDL-C, and LDL-C in liver; and fecal total BAs concentration were detected according to the instructions of commercial kits [Glu kit (A154-1-1), TG assay kit (A110-1-1), TC assay kit (A111-1-1), HDL-C assay kit (A112-1-1), LDL-C assay kit (A113-1-1), and BAs assay kit (E003-2-1)] (Nanjing Jiancheng Bioengineering Institute, Nanjing, China).

### 2.7 Histomorphology analysis

HE staining: Liver and abdominal adipose tissue samples were fixed, stained with hematoxylin for 5 min, washed with running water, dehydrated with gradient alcohol, and stained with eosin for 5 min. Oil Red O staining: Fresh frozen tissue sections were fixed and stained with Oil Red O. They were then immersed in 60% isopropyl alcohol for 5 s, followed by a hematoxylin counterstain for 5 min. The slides were fixed and observed under a light microscope.

### 2.8 RT-qPCR analysis

RNA was isolated from liver and ileum tissues by using TRIzol reagent (Invitrogen, CA, United States), as described in our previous study (Nie et al., 2015). RT-qPCR analysis was conducted using a Light Cycler 96 System (Roche Applied Science, Penzberg, Germany) with primers listed in Table 2, and *β-actin* was used as an internal control for the target genes. The 2^−ΔΔCt^ method was used to standardize the fold change in mRNA levels.

**Table 2:** A list of primer sequences used in this study.

Gene	Forward sequence (5′-3′)	Reverse sequence (5′-3′)	Gene Bank No.
*β-actin*	ATTGTCCACCGCAAATGCTTC	AAATAAAGCCATGCCAATCTCGTC	NM_205518.2
*FAS*	TCAGGGTGTTCTGGAATGCAA	AATCCTGGTGGGCAATCGTAG	NM_205155.4
*ACC*	AATCCTGGTGGGCAATCGTAG	GGAACATTCAGGATACGC	NM_205505.2
*CPT*	GCCCTGATGCCTTCATTCAA	ATTTTCCCATGTCTCGGTAGTGA	NM_001012898.1
*SREBP*	TCACCGCTTCTTCGTGGAC	CTGAAGGTACTCCAACGCATC	NM_204126.3
*LPL*	AGTCAGAGTGAAGTCAGGCGAAAC	CTGCTCCAGGCACTTCACAAATA	NM_205282.2
*PPARα*	TGCACTGGAACTGGATGATAGTGA	TCCTACATTTACAAGACCAGGACGA	NM_001001464.1
*PPARγ*	TGTGAAGTTCAACGCACTGGAATTA	GGAGCTCCAAAGCT TGCAACA	NM_001001460.2
*CYP7A1*	AGGTAACGCCCTAGATGC	GCACTGTGGGCACTCTT	NM_001001753.2
*CYP27A1*	CTGATGTCCCGACGCA	TCCGCATCGGGTATTT	XM_422056.8
*CYP8B1*	GGAGACGAAGACCCAATG	CTTACTTAGAAACCCTGAACTG	NM_001005571.1
*FXR*	ATGGGATCTGAAATGAATTTAAT	ATGCCAAATAAATGAGGAC	NM_001396910.1
*SHP*	CCCCAAAGAATATGCCTACC	TGTCTGCGTTGCCGATG	NM_001030893.3
*FGF19*	ATTCGTCCAGACGGCTACAA	GGGATCCATGCTGTCCGTTT	NM_204674.3
*IBABP*	ATGGCATTCACAGGCAAATATGA	TGCTGAGTCCAGGTGAA	NM_001277700.2
*ASBT*	ATGCAGTCTTACTTACTTTCTCG	AACTGACAGAGGAAACCC	NM_001319027.2

### 2.9 16S RNA sequencing

DNA was isolated from the samples using a DNA extraction kit (M5635-02) (Omega Bio-Tek, Norcross, GA, United States). The following steps were performed according to Niu’s method with slight modifications; details are provided in the Supplementary material (Niu et al., 2020).

### 2.10 LC/MS analysis of BAs

The liver tissue was homogenized, and BAs were extracted using a methanol extraction method, along with the addition of internal standards. Subsequently, the analysis was conducted on the BAs by using a combination of liquid chromatography and electrospray ionization tandem mass spectrometry; details are provided in the Supplementary material (Bhargava et al., 2020).

### 2.11 Lipidomics analysis

Liver tissue was homogenized, and lipids were extracted using a chloroform/methanol extraction method with the addition of internal standards. Subsequently, lipid analysis was conducted using a combination of liquid chromatography and electrospray ionization tandem mass spectrometry; details are provided in the Supplementary material (Werner et al., 2018).

### 2.12 Statistical analysis

One-way ANOVA was conducted using SPSS 22.0, significant differences were assessed using Duncan’s method, and correlation analysis was conducted using Spearman analysis. *p* < 0.05 indicates statistical significance, and *p* < 0.01 indicates high significance. All data are presented as mean ± SD. Data were entered into SPSS version 22.0, and correlation coefficients were calculated based on Spearman correlation distances. Heat maps were constructed using ORIGIN 2021 to assess the bivariate relationships between variables.

## 3 Results

### 3.1 Effect of ZD-3 on growth performance, fat deposition, and serum lipid indices in broilers

No significant differences were observed in Body weight, ADFI, ADG, and F/G among the three groups (Table 3). However, ZD and HZD treatments significantly reduced the abdominal fat index and subcutaneous fat thickness compared with the CON (*p* < 0.05) (Table 3). Treatment with ZD significantly reduced the serum GLU, TG, TC, and LDL-C levels relative to that of the CON (*p* < 0.05). Treatment with HZD resulted in a decreasing trend in TG and LDL-C levels relative to those of the CON. No significant differences were observed in the HDL-C levels among the three groups (Table 3).

**Table 3:** Effects of active and heat-inactivated ZD-3 on growth performance, fat deposition, and serum lipid indices in yellow-feathered broilers.

Items	Treatment			SEM	*p*-value
	CON	ZD	HZD		
Growth performance					
Body weight, g	701.98	692.86	694.30	7.418	0.878
ADG, g/d	23.46	23.38	23.45	0.262	0.991
ADFI, g/d	54.38	52.91	53.62	0.428	0.397
F/G	2.32	2.26	2.29	0.026	0.747
Fat deposition					
Abdominal fat index, %	1.43a	0.79b	0.92b	0.088	0.002
Subcutaneous fat thickness, cm	0.17a	0.11b	0.09b	0.010	0.003
Serum lipid indices					
GLU, mmol/L	12.48a	9.80b	10.88b	0.374	0.009
TC, mmol/L	3.78a	2.83ab	3.61b	0.167	0.019
TG, mmol/L	0.52a	0.35ab	0.44b	0.026	0.034
LDL-C, mmol/L	0.80a	0.53ab	0.63b	0.047	0.050
HDL-C, mmol/L	1.76	1.90	1.96	0.231	0.944

^ab^ indicates significant differences compared to CON at *p* < 0.05, respectively.

### 3.2 Effect of ZD-3 on tissue morphology, liver lipid indices, and lipid gene expression in broilers

**Figure 1:**
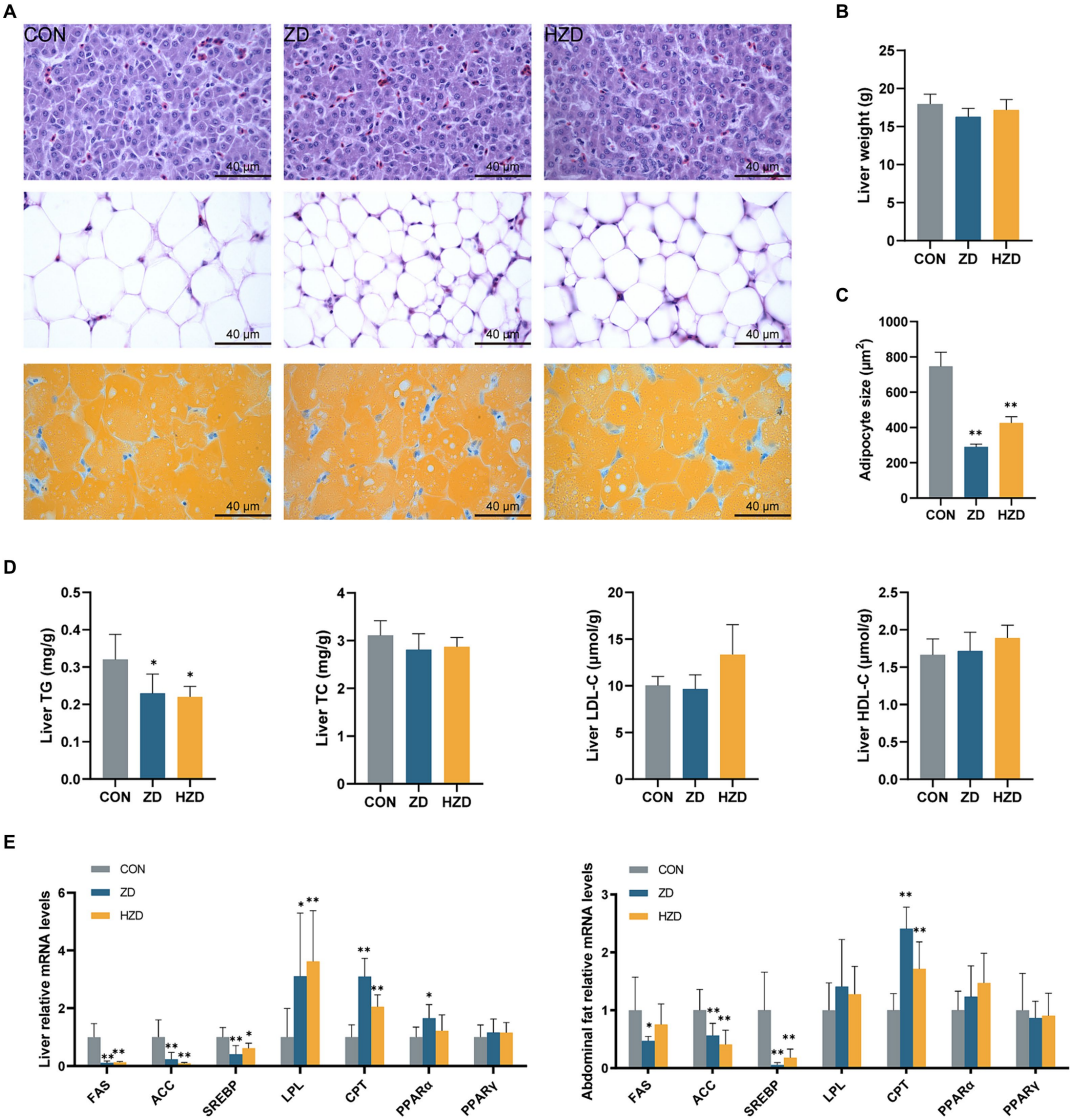
Effects of active and heat-inactivated ZD-3 on liver and abdominal fat metabolism in yellow-feathered broilers. **(A)** Representative images of H/E staining and Oil Red O staining of liver and abdominal fat sections (100 ×, scale bar = 40 μm). **(B)** Liver weight. **(C)** Distribution of adipocyte size in abdominal fat. **(D)** Liver lipid parameters indices: GLU, TG, TC, HDL-C, and LDL-C. **(E)** Lipid-related gene expression in the liver and abdominal fat (FAS, fatty acid synthase; ACC, acetyl CoA carboxylase; SREBP, sterol-regulatory element-binding protein 1c; LPL, lipoprotein lipase; CPT, carnitine palmitoyl transferase; PPAR-α, peroxisome proliferator-activated receptor-α; PPAR-γ, peroxisome proliferator-activated receptor-γ). Values are represented as means ± SD (*n* = 6). *p < 0.05, **p < 0.01 and indicate the significant differences between the CON.

HE staining revealed that of the CON, the hepatic cell arrangement was lax and chaotic, with numerous fat vacuoles of varying sizes and quantities. When broilers were treated with ZD or HZD, the fat droplets were reduced (Figure 1A). No significant differences were observed in the liver weight among the three groups (Figure 1B). The ZD and HZD treatments significantly decreased the average size of abdominal adipocytes relative to that in the CON (*p* < 0.05) (Figure 1C) and significantly reduced the liver TG levels relative to those in the CON (*p* < 0.05). No significant differences were observed in the TC and HDL-C levels among the three groups (Figure 1D). The ZD and HZD treatments significantly increased the levels of lipoprotein lipase (LPL) and carnitine palmitoyl transferase (CPT) and decreased the levels of fatty acid synthase (FAS), acetyl CoA carboxylase (ACC), and sterol-regulatory element-binding protein 1c (SREBP) in the liver compared with those of the CON (*p* < 0.05). Treatment with ZD and HZD significantly decreased the levels of ACC and SREBP in abdominal fat compared with those of the CON (*p* < 0.05) (Figure 1E).

### 3.3 Effect of ZD-3 on ileal microbiota structure in broilers

**Figure 2:**
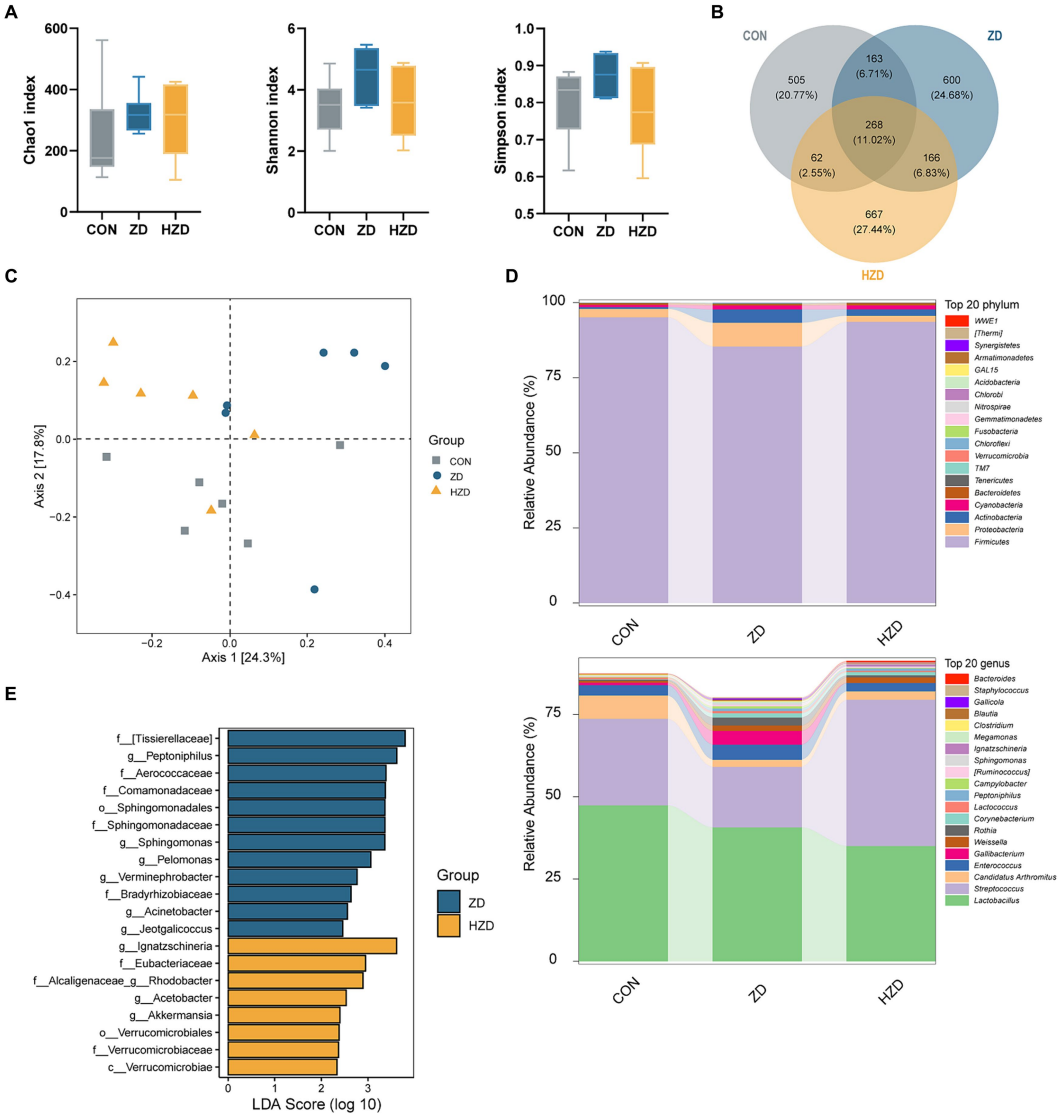
Effects of active and heat-inactivated ZD-3 on ileal microbiota structure in yellow-feathered broilers. **(A)** Alpha-diversity assessed using richness (Chao1 index) and diversity (Shannon index and Simpson index). **(B)** Venn diagram of OTUs among the groups. **(C)** Beta diversity calculated using Bray-Curtis based PCoA. **(D)** Relative abundance of microbiota at the phylum and genus level. **(E)** LDA score (LDA > 2) among the groups. Values are represented as means ± SD (*n* = 6).

A comprehensive analysis of the broiler ileum microbiota using 16S rRNA sequencing technology revealed the presence of 2,431 operational taxonomic units (OTUs), comprising 21 phyla, 51 classes, 87 orders, 165 families, 346 genera, and 467 species. The α diversity of the ileum microbiota in broilers exposed to different treatments was assessed using OTU levels. The Chao1 index of the ileal microbiota of the ZD and HZD was higher than that of the CON, and the Shannon and Simpson indices of the ileal microbiota of the ZD group were higher than those of the CON and HZD groups (Figure 2A). The ZD and HZD treatments resulted in a higher abundance of ileum microbiota in broilers, with an increase in the number of OTUs by 19.94 and 16.53%, respectively, than that of the CON (Figure 2B). The principal coordinate analysis (PCoA) of β diversity revealed that the ileum microbiota of the ZD and HZD was similar but different from that of the CON (Figure 2C). Additional analysis of the composition of ileum microbiota at the phylum level revealed that the ZD and HZD treatments decreased the relative abundance of *Firmicutes* by 10.23 and 1.60%, respectively, and significantly increased the relative abundance of *Bacteroidetes* by 231.85 and 325.99%, respectively (*p* < 0.05), decreasing in the F/B ratio compared with that of the CON (Figure 2D). Additional analysis of the composition of the ileum microbiota at the genus level revealed that the ZD treatment increased the abundance of *Enterococcus*, *Gallibacterium*, *Weissella*, and *Rothia* compared with that of the CON (Figure 2D). LEfSe analysis revealed significant differences in the abundances of 20 species among the three groups, with 12 species being most abundant in the ZD group and eight species in the HZD group (Figure 2E).

### 3.4 Effect of ZD-3 on liver BA profile and enterohepatic circulation of BAs in broilers

**Figure 3:**
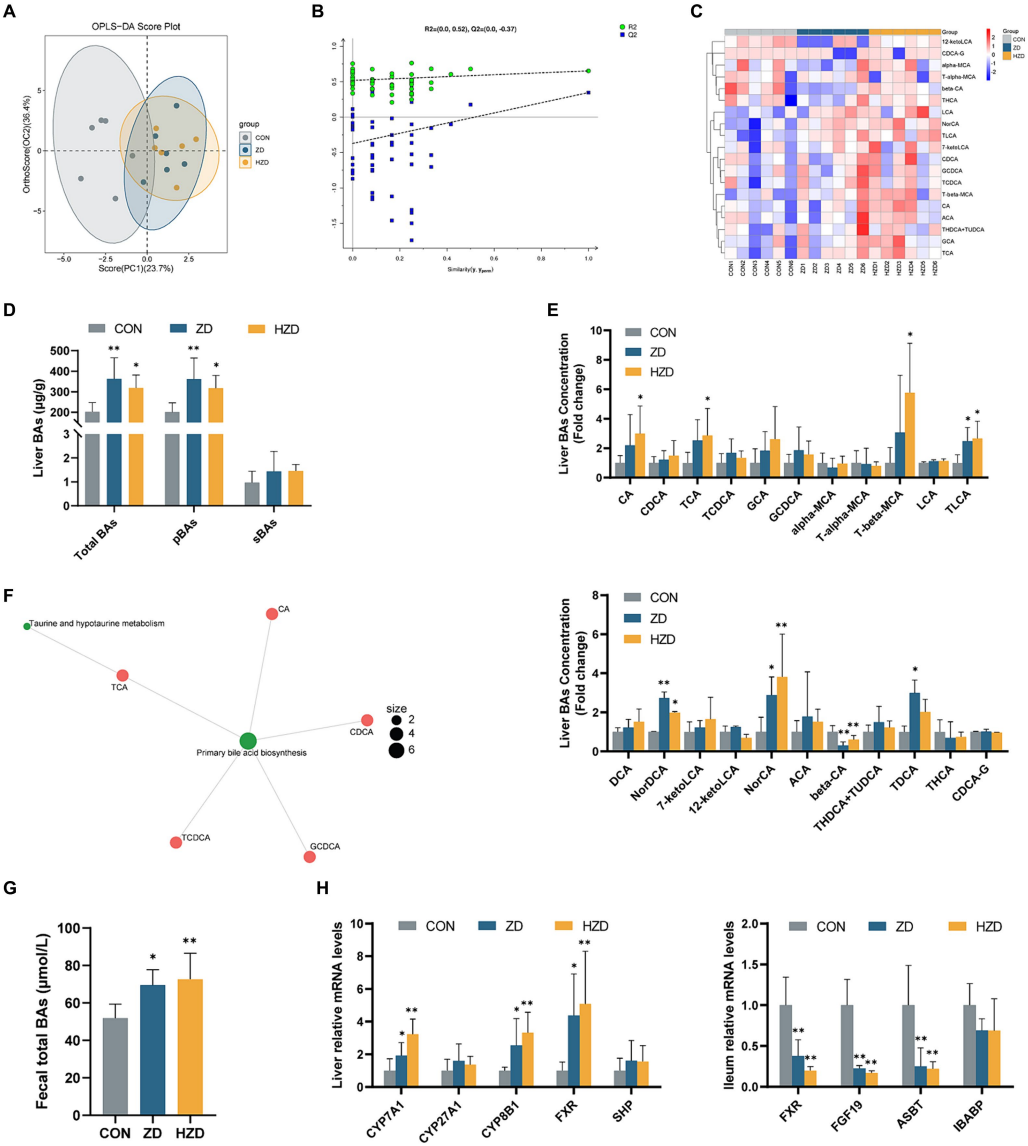
Effects of active and heat-inactivated ZD-3 on the enterohepatic circulation of BAs in yellow-feathered broilers. **(A)** Orthogonal partial least squares-discriminant analysis (OPLS-DA) based on the liver BAs metabolites. **(B)** Corresponding validation plots based on the liver BAs metabolites. **(C)** Hierarchical clustering heatmaps of the BAs contents in liver among the groups. **(D)** Liver BAs concentration: total BAs; pBAs, primary BAs; sBAs, secondary BAs. **(E)** Levels of BAs in the liver. **(F)** KEGG metabolite molecular network diagram based on the liver BAs metabolites. **(G)** Fecal total BAs concentration: total BAs. **(H)** BAs-related gene expression in the liver and ileum (CYP7A1, cytochrome P450 family 7 subfamily A member 1; CYP27A1, cytochrome P450 family 27 subfamily A member 1; CYP8B1, steps sterol 12-α-hydroxylase; FXR, farnesoid x receptor; SHP, small heterodimer partner; FGF19, fibroblast growth factor 19; ASBT, apical sodium-dependent BA transporter; IBABP, ileum BA binding protein). Values are represented as means ± SD (*n* = 6). *p < 0.05, **p < 0.01 and indicate the significant differences between the CON.

OPLS-DA was used to identify clustering trends in the data. The results of the ZD and HZD differed slightly from those of the CON (Figure 3A). A permutation test further validated the reliability of the model (Figure 3B). Hierarchical clustering heatmaps of the BA content among the three groups showed that when broilers were treated with ZD and HZD, the liver BA profile differed from that of the CON (Figure 3C). The liver BA change revealed that the ZD and HZD treatments significantly increased the concentration of total BAs and primary BAs relative to that of the CON (*p* < 0.05) (Figure 3D). The ZD and HZD treatments significantly increased the levels of Norcholic acid (NorCA), Nordeoxycholic Acid (NorDCA), and Taurolithocholic acid sodium salt (TLCA) compared with those of the CON but decreased the 3β-Cholic acid (β-CA) levels (*p* < 0.05). The ZD treatment significantly increased the levels of Tauro deoxycholic acid sodium salt (TDCA), and the HZD treatment significantly increased the levels of Cholic acid (CA), Taurocholic acid Sodium Salt (TCA), and Tauro-β-muricholic acid Sodium Salt (T-β-MCA) compared with those of the CON (*p* < 0.05) (Figure 3E). In this study, BAs from the three groups of samples were combined for KEGG pathway enrichment analysis for the biological interpretation of higher-level system functions. The metabolites identified using non-targeted metabolomics were primarily associated with the biosynthesis of primary BAs and taurine and hypo taurine metabolism (Figure 3F). The ZD and HZD treatments significantly increased the concentration of total BAs in the fecal matter compared with that of the CON (*p* < 0.05) (Figure 3G). ZD and HZD significantly increased the levels of cholesterol 7-α-hydroxylase (CYP7A1), sterol 12-α-hydroxylase (CYP8B1), and FXR in the liver and decreased the levels of FXR, FGF19, and apical sodium-dependent BA transport (ASBT) in the ileum compared with those of the CON (*p* < 0.05) (Figure 3H).

### 3.5 Effect of ZD-3 on liver lipid composition in broilers

**Figure 4:**
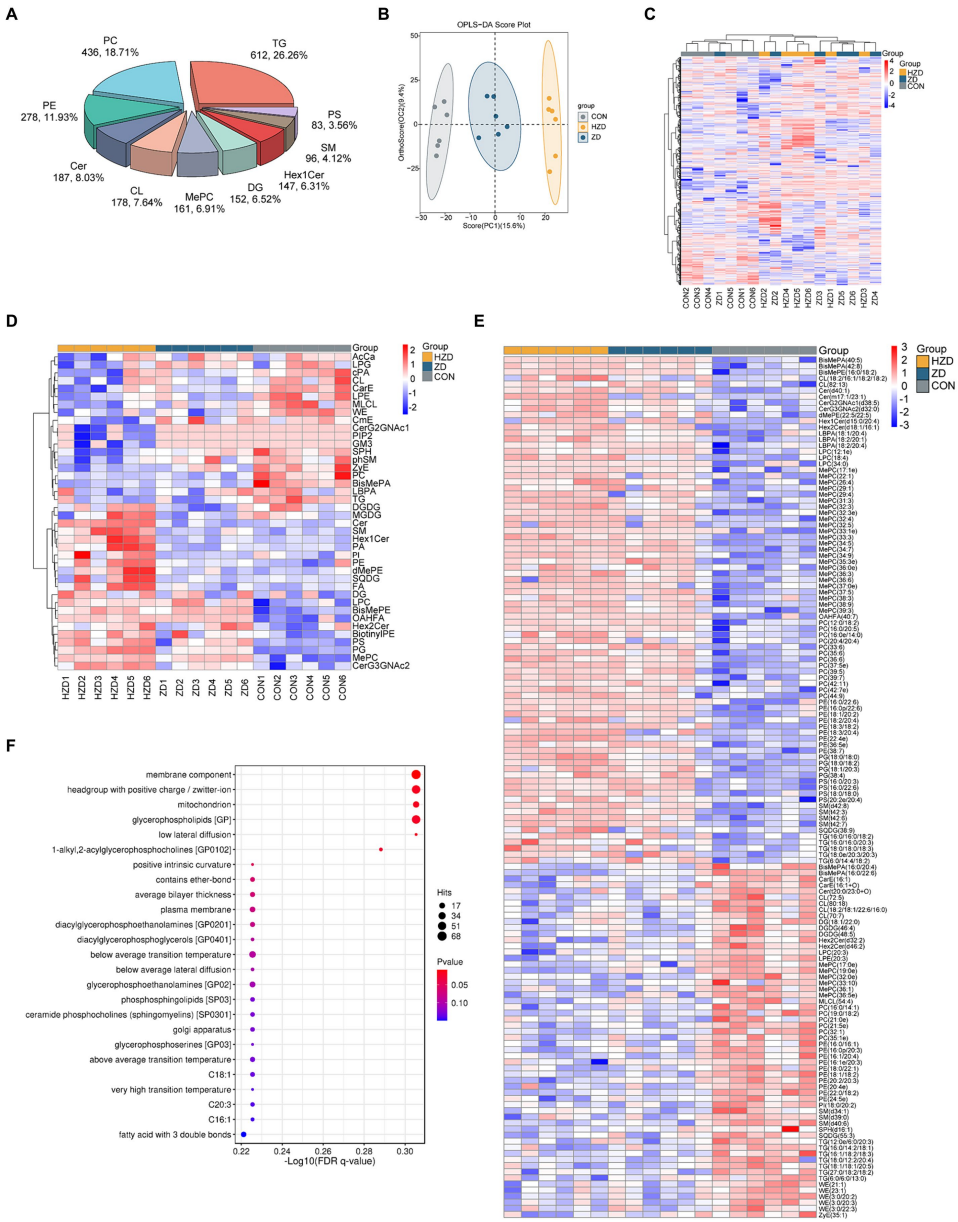
Effects of active and heat-inactivated ZD-3 on liver lipid composition in yellow-feathered broilers. **(A)** Percentages of the top 10 different lipid classes. **(B)** OPLS-DA based on the liver lipid metabolites. **(C)** Heatmaps based on total liver lipid content. **(D)** Hierarchical clustering heatmaps based on liver lipid classes. **(E)** Heatmaps of liver differential abundance (DA) of individual lipids (abundance increased more than 2.5 times and decreased more than 1.5 times compared with CON). **(F)** Lipid metabolic pathway analysis based on the liver lipid metabolites. The color indicates the significance of the enrichment *p* value, and the redder the color, the more significant the enrichment result; the size of the bubble point indicates the number of differential lipids enriched to the item, and the larger the bubble point, the more the enriched lipids are (*n* = 6).

A total of 3,229 lipid metabolites were detected in liver tissues across the three groups, namely 612 TG, 436 Phosphatidylcholine (PC), 278 Phosphatidylethanolamine (PE), 187 Ceramides (Cer), 178 Cardiolipin (CL), 161 methylated phosphatidylcholine (MePC), 152 Diglyceride (DG), and 147 Hexosylceramides (Hex1Cer) (Figure 4A). The control and treatment groups were distinguished using the OPLS-DA results (Figure 4B). However, the ZD and HZD groups could not be distinguished (Figure 4C). Heat map visualization revealed a significant decrease in the relative abundances of TG, PC, Lyso-phosphatidylethanolamine (LPE), and Zymosteryl (ZyE) and a significant increase in the relative abundances of Phosphatidylglycerol (PG), Lyso-phosphatidylcholine (LPC), and Phosphatidylserine (PS) in the ZD and HZD groups compared with the CON (*p* < 0.05) (Figure 4D). A total of 141 differential abundance (DA) lipids were identified (abundance increased more than 2.5 times and decreased more than 1.5 times compared with CON). Heat map visualization (Figure 4E) confirmed that the individual lipids in the liver could be clearly classified into three groups, which was consistent with the analyses shown in Figure 4D. In this study, DA lipids from the three groups of samples were combined for lipid functional enrichment analysis, lipid classification, chemical and physical properties, and function and subcellular component interpretation of the higher-level system functions. We found that the metabolites identified through non-targeted metabolomics were primarily associated with membrane components, headgroups with positive charges/zwitterions, mitochondria, and glycerophospholipids (GP) (Figure 4F).

### 3.6 Correlation of ileal microbiota structure, liver BA profile, liver lipid composition, and fat deposition in broilers

**Figure 5:**
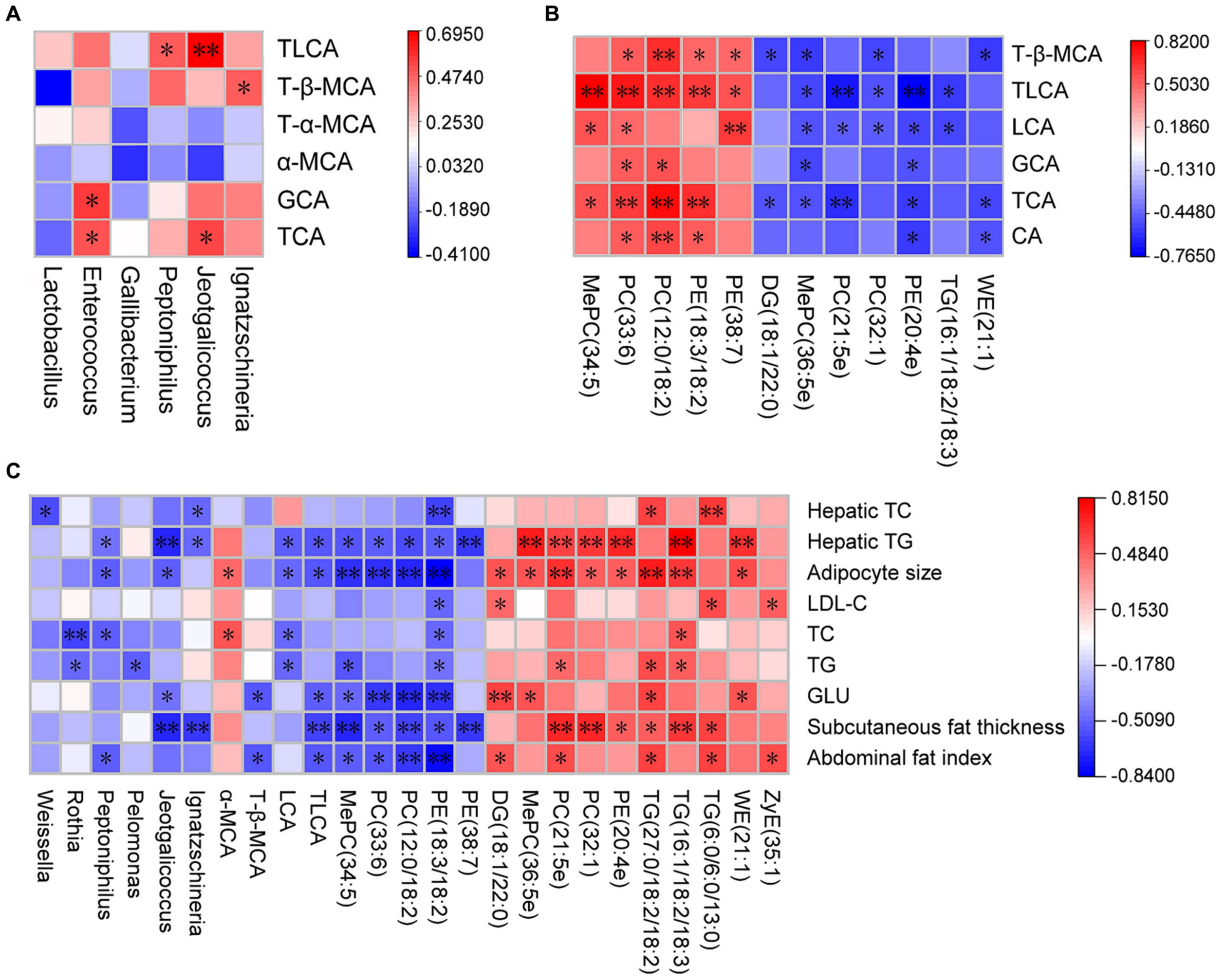
Correlation of ileal microbiota structure, liver BA profile, liver lipid composition and fat deposition in active and heat-inactivated ZD-3-treated broilers. **(A)** Correlation between ileal microbiota structure and liver BA profile. **(B)** Correlation between liver BA profile and liver lipid composition. **(C)** Correlation between ileal microbiota structure, liver BA profile, liver lipid composition, and fat deposition. Cells are colored based on Spearman’s correlation coefficient: red represents a negative correlation, and blue represents a positive correlation, *p < 0.05, **p < 0.01.

Further analysis of Pearson correlations showed correlations among ileal microbial community, liver BA profile, liver lipid composition, and fat deposition. TCA had significant positive correlations with *Jeotgalicoccus* and *Enterococcus*. glycocholicacid (GCA) had significant positive correlations with *Enterococcus* (*p* < 0.05). T-β-MCA had significant positive correlations with *Ignatzschineria* (*p* < 0.05). TLCA had significant positive correlations with *Peptoniphilus* and *Jeotgalicoccus* (*p* < 0.05) (Figure 5A). MePC (34.5) had significant positive correlations with TCA, Lithocholic acid (LCA), and TLCA (*p* < 0.05). PC (33:6) and PC (12:0/18:2) had significant positive correlations with CA, TCA, GCA, TLCA, and T-β-MCA (*p* < 0.05). PE (18:3/18:2) had significant positive correlations with CA, TCA, TLCA, and T-β-MCA (*p* < 0.05). PE (38:7) had significant positive correlations with LCA, TLCA, and T-β-MCA (*p* < 0.05). DG (18:1/22:0) had significant negative correlations with TCA and T-β-MCA (*p* < 0.05). MePC (36:5e) and PE (20:4e) had significant negative correlations with TCA, GCA, LCA, and TLCA (*p* < 0.05). PC (32:1), PC (21:5e), and TG (16:1/18:2/18:3) had significant negative correlations with LCA and TLCA (*p* < 0.05). WE (21:1) had significant negative correlations with CA, TCA, and T-β-MCA (*p* < 0.05) (Figure 5B). The abdominal fat index had significant negative correlations with *Peptoniphilus*, TLCA, MePC34.5, PC (33:6), PC (12:0/18:2), and PE (18:3/18:2) and positive correlations with DG (18:1/22:0), PC (21:5e), TG (27:0/18:2/18:2), TG (6:0/6:0/13:0), and ZyE (35:1) (*p* < 0.05). Subcutaneous fat thickness had significant negative correlations with *Jeotgalicoccus*, *Ignatzschineria*, TLCA, MePC34.5, PC (33:6), PC (12:0/18:2), PE (18:3/18:2), and PE (38:7) and positive correlations with PC (21:5e), PC (32:1), PE (20:4e), TG (16:1/18:2/18:3), TG (27:0/18:2/18:2), and TG (6:0/6:0/13:0) (*p* < 0.05) (Figure 5C).

## 4 Discussion

The results of the present study showed a decrease in broiler body weight and F/G under the influence of *Saccharomyces cerevisiae* tropicalis, which is consistent with the previous study (Niu et al., 2019). Probiotics prevent excessive abdominal fat deposition by inhibiting lipid synthesis and promoting fatty acid decomposition (Wang et al., 2022). In addition, ZD and HZD treatments reduced subcutaneous fat thickness and abdominal fat percentage, which suggests that ZD-3 can regulate fat metabolism in broilers, and heat-inactivated ZD-3 can play the same role and explain the loss of body weight in broilers. ZD-3 could reduce abdominal fat deposition and F/G without affecting other growth performance in broilers, thereby enhancing economic efficiency. Therefore, ZD-3 has the potential to be a novel probiotic preparation with lipid-lowering effects. This is also evidenced by the serum lipid indices. In this study, under the influence of ZD-3, GLU enters tissue cells for oxidative catabolism to provide energy and reduce fat deposition in broilers through energy metabolism. TG in broilers is mainly stockpiled in the abdominal fat, and serum TG levels and abdominal fat usually decrease simultaneously (Kim and Voy, 2021). The results showed that the addition of ZD-3 effectively reduced serum TG levels. To some extent, probiotics play a role in lowering serum TC levels (Qu et al., 2020). Meanwhile, ZD-3 has a beneficial effect on lowering serum TC and LDL-C, and the underlying mechanism may be that its cell walls adsorb cholesterol, or the bacterial cells transport cholesterol out of the body, promoting lipid metabolism (Ooi and Liong, 2010).

Hepatocytes are the major parenchymal cells of the liver that play a crucial role in the maintenance of liver and systemic lipid metabolism (Zhou et al., 2022). In this study, ZD-3 reduced the liver weight and intracellular lipid vacuoles. In this study, the surface area of adipocytes in abdominal fat was reduced in broilers fed ZD-3. Thus, ZD-3 can inhibit the enlargement of fat cells, leading to a decrease in fat accumulation. Consistent with the serum lipid results, the TG level in the liver of broilers fed ZD-3 was also significantly reduced. The ZD-3 strain demonstrated significant lipid-lowering effect, confirmed by serum and liver lipid indices and histomorphology tests. In general, cell viability is the main factor affecting the function of probiotics (Brandão et al., 2021). Our results indicate that heat-inactivated ZD-3 retains some physiological activity to provide lipid-lowering benefits to the host.

Additionally, ZD-3 regulated the expression of genes involved in lipid metabolism in broilers. Peroxisome proliferator-activated receptor-α (PPAR-α) is highly expressed in broiler liver and is primarily involved in the regulation of fatty acid oxidation and glucose metabolism (Gu et al., 2022). CPT is a major regulator in the control of fatty acid β-oxidation in mitochondria (Dai et al., 2018). The mRNA levels of PPARα and CPT were increased in broilers fed with ZD-3, which may lead to a reduction in fat deposition by promoting fatty acid oxidation in the liver. LPL is a key enzyme that hydrolyzes TG to release fatty acids and glycerol (Huang et al., 2023). The increased expression of the LPL gene in the livers of broilers fed ZD-3 indicates that it may promote lipolysis, which is corroborated by the decrease in TG levels in the liver. SREBP and its response genes ACC and FAS activate fatty acid (FA) biosynthesis (Currie et al., 2013). In addition, ZD-3 reduced the mRNA expression of SREBP, ACC, and FAS in abdominal fat and liver tissue, implying that it reduces FA synthesis and fat deposition. Our study suggests that ZD-3 reduces the accumulation of fat in the liver and abdominal fat tissue by decreasing the mRNA expression related to *de novo* FA synthesis and increasing the mRNA expression related to FA oxidation.

It has been shown that probiotic supplements can effectively change the composition of the intestinal microbiota and influence its regulation of lipid metabolism, preventing the accumulation of abdominal fat (Helmink et al., 2019). Our study unequivocally demonstrated that ZD-3 led to changes in the composition of ileal microbial communities and an increase in microbial diversity in broilers. The principal microbial communities in the ileum of the broilers were *Firmicutes* and Proteobacteria (He et al., 2019). An increased abundance of *Firmicutes* usually disturbs host energy metabolism and results in fatty liver disease. The addition of ZD-3 led to a reduction in the abundance of *Firmicutes* in the ileum, which may lead to a decrease in liver lipids. In addition, ZD-3 increased the abundance of *Bacteroidetes* and decreased the F/B ratio, which has traditionally been considered an indicator of obesity. In this study, the addition of ZD-3 to the diet did not alter the dominance of *Firmicutes* in the ileum but increased the relative abundance of *Bacteroidetes* to regulate fat deposition. At the genus level, the abundance of *Lactobacillus*, *Streptococcus*, *Enterococcus*, *Gallibacterium*, *Weissella*, and *Rothia* underwent significant changes in this study, which have been documented as potentially related to lipid metabolism (Turnbaugh et al., 2009). There are reports that *Enterococcus* has good cholesterol-lowering activity, which may improve lipid metabolism and prevent liver damage (Yang et al., 2021). In this study, ZD-3 increased the abundance of *Enterococcus*, which helped regulate cholesterol content to reduce fat deposition. It has been shown that mice fed a high-fat diet have a significantly reduced gut microbiota of *Weissella* (Liu et al., 2021), whereas its abundance was significantly increased by feeding ZD-3. In addition, *Rothia* has been enriched in the ZD group, which is beneficial to the host and contributes to regulating GLU metabolism, reducing lipid levels, and improving inflammation (Wang M. et al., 2023). These bacteria can produce bile salt hydrolase (BSH), a crucial component of the interaction between microbes and the host, which regulates the metabolism of host cholesterol and BAs (Liu et al., 2023). Notably, the alteration of the BA profile induced by BSH-producing bacteria may have a significant impact on promoting lipolysis. In addition, the microbial composition related to lipid metabolism was identified through LEfSe analysis. *Sphingomonas* synthesizes sphingosine, which subsequently combines with FA derivatives to form sphingomyelin, decreasing lipid accumulation in the liver (Zhang X. et al., 2022). *Jeotgalicoccus* produces cytochrome P450 enzymes for the decarboxylation and hydroxylation of fatty acids, which affects their levels in the host organism (Rude et al., 2011). *Acetobacter* synthesizes acetyl coenzyme A synthetase, which regulates the tricarboxylic acid cycle (Guertin and Wellen, 2023). These results suggest that active and heat-inactivated ZD-3 may improve host lipid metabolism by altering the composition and function of the gut microbiota and possibly by affecting the enterohepatic circulation of BA.

In the liver, cholesterol is primarily excreted from the body by being converted into bile acids. Cholesterol is converted to BAs and secreted into the ileum to facilitate the absorption of dietary lipids. Enterohepatic circulation of BAs is a highly efficient physiological mechanism (Chambers et al., 2019). Our research suggests that ZD-3 altered the liver BA profile and increased the relative concentration of BAs and the levels of CA, TCA, and GCA in liver tissue. These changes may have been influenced by alterations in the ileal microbiota (Collins et al., 2023). Spearman analysis revealed statistically significant positive correlations between TCA and the bacterial species *Enterococcus* and *Jeotgalicoccus*, and GCA had significant positive correlations with *Enterococcus*. ZD-3 has been found to contribute to the conversion of cholesterol into primary BA, and changes in BA profile were mainly reflected in the enrichment of taurine-conjugated BAs. Taurine metabolism prevents obesity by modulating mitochondrial function and promoting browning of adipose tissue (Guo et al., 2019). In this study, primary BA were primarily released as taurine-conjugated BAs in the ileum to activate the enterohepatic axis and reduce liver cholesterol levels. To gain insight into the process of BAs enterohepatic circulation, we analyzed the gene expression in broilers. In this study, the liver mRNA levels of CYP7A1 and CYP8B1 were significantly increased in broilers fed ZD-3. This result suggests that ZD-3 mainly enhances the classical pathway, accelerating the conversion of cholesterol. As CA is an activator of FXR, FXR expression was increased in the liver of broilers fed with ZD-3. When FXR is activated, it induces downstream FGF19 release, which regulates BAs metabolism (Jia et al., 2023). FXR activation down-regulates the expression of the target gene FAS and inhibits the expression of SREBP via the small heterodimer partner (SHP) and stimulates the expression of PPARα and peroxisome proliferator-activated receptor-γ (PPARγ), indirectly promoting the entry of fatty acids into mitochondria for β-oxidation (Shen et al., 2011; Xu et al., 2022). These results suggest that FXR has a significant impact on the regulation of lipid metabolism in the liver. The substantial buildup of taurine-conjugated BAs in the intestine can alleviate the occurrence of fatty liver by suppressing intestinal FXR (Huang et al., 2019). In addition, the production of BSH by the gut microbiota leads to an increase in the excretion of unconjugated BAs in the faces, facilitating the conversion of cholesterol into BAs (He and You, 2020). In our study, ZD-3 suppressed the FXR-FGF19 signaling pathway in the intestine by increasing the abundance of BSH-producing microbiota and promoting the formation of taurine-conjugated BAs. In the ileum, 95% of BAs are absorbed into the liver in the presence of ASBT and ileal BA binding protein (IBABP), completing enterohepatic circulation and regulating the size of the BA pool (Al-Dury and Marschall, 2018). ASBT inhibitors have been shown to improve hepatic steatosis in mice by modulating BA metabolism (Rao et al., 2016). In this study, ZD-3 treatment resulted in the downregulation of ileum ASBT expression, leading to increased BAs elimination, and decreased liver cholesterol levels. As a result, ZD-3 may reduce cholesterol accumulation in the liver and regulate lipid metabolism through a combination of decreased ileal FXR-FGF19 signaling via the gut microbiota-BA-FXR pathway and increased liver FXR-SHP signaling. Heat-inactivated ZD-3 could play the same role, possibly because the cell wall, outer membrane proteins, and metabolites of ZD-3 retain some biological activity.

In this study, we found that ZD-3 induced changes in liver lipid biomarkers, which may explain the reduction in blood lipid levels and abdominal fat deposition. Significant but variable changes were observed in glycolipids (TG and DG), phospholipids (PC, PE, PG, and PS), and sphingolipids (Cer and Hex1Cer). The lipid classes may play a crucial role in regulating lipid metabolism in the liver of ZD-3. Of the 141 lipid molecules screened, downregulated lipids mainly belonged to the TG, DG, and Carboxylesterase (CarE) classes. The reduction in liver TGs was due to changes in the levels of the LPL, SREBP, ACC, and FAS genes. In hepatocytes, the reduction of CarE results in a decreased rate of lipid movement toward preexisting cytoplasmic lipid droplets, leading to a reduction in their size. This effect reduced weight gain, lowered blood lipid levels, and improved fatty liver disease (Lian et al., 2018). GP are the most abundant phospholipids found in living organisms. They form biofilms, are components of bile and membrane surfactants, and are involved in the recognition and signaling of proteins by cell membranes (Wang et al., 2020). We found multiple altered GP components in broilers fed ZD-3, such as PC, PE and MePC. PC, a major component of cell membranes, is involved in the secretion of very-low-density lipoproteins in the liver. Abnormally low or high levels can lead to steatosis (Kim et al., 2018). Lipolysis of the PE class was the main cause of the increase in free fatty acids. MePC is a methylated phospholipid and an important precursor of PC formation. Heatmap visualization showed that PC (33:6), PC (12:0/18:2), PC (32:1), PC (21:5e), PE (18:3/18:2), PE (38:7), PE (16:0/16:1), PE (20:4e), MePC (34:5), and MePC (36:5e) may be key lipids that mediate cell membrane signal transduction to regulate liver lipid metabolism. Spearman’s analysis suggested that elevated levels of liver CA, TCA, GCA, LCA, and TLCA caused the GP changes in liver tissue.

Sphingolipids (SP) are components of cell membranes and lipoproteins involved in intracellular and extracellular signal transduction and the induction or activation of genes that regulate lipid metabolism (Summers et al., 2019). Cer serves as the central component of the SP pathway and can be converted into Sphingomyelin (SM) and HexCer. SM is a primary constituent of cell membranes. Changes in the levels of SM molecules in the cell membrane cause corresponding changes in cholesterol levels (St Clair et al., 2020). Thus, these altered SP might also play a role in regulating liver lipid metabolism by ZD-3. We also found higher levels of WE (21:1), WE (23:1), and ZyE (35:1) in the CON, and these DA lipids might be markers of lipid accumulation. The DA lipids examined in this study had various physiological functions, either beneficial or detrimental to lipid metabolism, and mainly belonged to the classes of PC and TG. Therefore, ZD-3 may regulate liver fat deposition by promoting TG degradation and modulating signal transduction in the cell membrane.

The liver is the center of fat transport. Changes in liver lipids in broilers can impact alterations in abdominal fat lipids and potentially influence systemic lipid changes. In this study, ZD-3 reduced abdominal fat deposition by altering the structure of ileal microbiota, liver BA profile, and liver lipid composition, and *Jeotgalicoccus*, *Ignatzschineria*, TCA, TLCA, MePC (34:5), PC (12:0/18:2), PE (18:3/18:2), and TG (16:1/18:2/18:3) may be markers for avoiding excessive fat deposition.

## 5 Conclusion

The addition of ZD-3 to feed helps prevent excessive fat deposition in yellow-feathered broilers by influencing the composition of *Firmicutes* and *Bacteroidetes* in the ileum microbiota, inhibiting ileal FXR-FGF19 and activating the liver FXR-SHP signaling pathway, which promotes the conversion of cholesterol to BAs and its excretion in the feces, and altering the liver lipid composition of TG and PC classes.

## Data Availability

The datasets presented in this study can be found in online repositories. The names of the repository/repositories and accession number(s) can be found below: https://www.ncbi.nlm.nih.gov/, PRJNA1072686.
