# Supplementary material for: Candida tropicalis ZD-3 prevents excessive fat deposition by regulating ileal microbiota and bile acids enterohepatic circulation in broilers
Source: Front Microbiol. 2024 Aug 14;15:1419424. doi: 10.3389/fmicb.2024.1419424 (PMC11353401; doi:10.3389/fmicb.2024.1419424)
Supplement: Supplementary file 1 [file Table_1.DOCX]

Supplementary Material

# 16S RNA sequencing

DNA was extracted from the samples using a DNA extraction kit (M5635-02) (Omega Bio-Tek, Norcross, GA, USA). After passing the test by 1% agarose gel electrophoresis, the V3 – V4 region of the 16S rDNA of the samples was PCR-amplified using the universal primers 338F (5′-ACTCCTACGGGAGGCAGCAG-3′) and 806R (5′-GGAC TACHVGGGTWTCTAAT-3′). After the individual quantification step, amplicons were pooled in equal amounts and pair-end 2×250 bp sequencing was performed using the Illumina NovaSeq platform with NovaSeq 6000 SP Reagent Kit (500 cycles) at Suzhou PANOMIX Biomedical Tech Co. LTD. Microbiome bioinformatics were performed with QIIME2 2019.4 according to the official tutorials (https://docs.qiime2.org/2019.4/tutorials/). OTUs (operational taxonomic units) were clustered using the UCLUST (version 7.1, http://drive5.com/uparse/). The representative sequences were aligned against Greengenes 13.5 using PyNAST and the default parameters were set by QIIME, and then alpha diversity analysis and principal coordinate analysis (PCoA) were conducted using the QIIME2 software. LEfSe (Linear discriminant analysis effect size) was performed to detect differentially abundant taxa across groups using the default parameters.

# LC/MS analysis of BAs

The hepatic samples were extracted in 400 μl of methanol (−20 ℃) with two steel balls and vortexed for 60 s. Then, the samples were added into a tissue grinder, ground at 55 Hz for 1 min, and the above operation was repeated at least twice. The samples were sonicated for 30 min at room temperature, centrifuged at 12,000 rpm and 4 ℃ for 10 min, and 300 μl of the supernatant was taken and 600 μl of water was added and vortexed for 30 s. The supernatant was filtered through 0.22-μm membrane, and the filtrate was added to the LC-MS. The gas chromatography conditions: ACQUITY UPLC® BEH C18 column (2.1×100 mm, 1.7μm, Waters, USA) were used; the injection volume was 5 μL, the column temperature was 40 ℃, and the mobile phase was A-0.01 % formic acid water and B-acetonitrile.

# Lipidomics analysis

Lipid extraction: Approximately 50 mg of hepatic sample in a 2 mL centrifuge tube was taken, 750 μl of mixed solvent (chloroform : methanol, 2:1, v/v) was added, and vortexed for 30 s; steel balls were added, added into the tissue grinder, and ground for 60 s at 50 Hz; this was repeated twice and the tube was left on ice for 40 min, 190 μl H2O was added, and vortexed for 30 s; the sample was incubated on ice for 10 min, centrifuged at 12000 rpm for 5 min at room temperature, and 300 μl of organic layer was transferred into a novel centrifuge tube, to which 500 μl of mixed solvent (chloroform : methanol, 2:1, v/v) was added and vortexed for 30 s. This was followed by centrifugation at 12000 rpm for 5 min at room temperature and then 400 μL of organic layer was transferred into the same centrifuge tube. Samples were concentrated to dry in vacuum and then dissolved in 200 μl isopropanol, and the supernatant was filtered through a 0.22-µm membrane to obtain the prepared samples for LC-MS. Chromatographic separation was done using an ACQUITY UPLC® BEH C18 (2.1 × 100 mm, 1.7 µm, Waters) column maintained at 50 ℃. The temperature of the autosampler was 8 ℃. Gradient elution of analytes was carried out with acetonitrile: water = 60 : 40 (0.1% formic acid +10 mM ammonium formate) (A2) and isopropanol : acetonitrile = 90 : 10 (0.1% formic acid +10 mM ammonium formate) (B2) at a flow rate of 0.25 mL/min.

# Chemicals, reagents and Instruments

LC-MS grade isopropyl alcohol (IPA) and methanol (MeOH) were purchased from Fisher Scientific (Loughborough, UK). Chloroform was obtained from sinopharm (Shanghai, China). Ultrapure water was generated using a Milli-Q system (Millipore, Bedford, USA). High-speed centrifuge was obtained from Hunan Xiangyi Experiment Equipment Co., Ltd. (Hunan, China). Vortex mixer was obtained from Haimen Kylin-bell Lab Instruments Co., Ltd. (Haimen, China). Centrifugal vacuum evaporator was from Eppendorf China Ltd. (Shanghai, China). Tissue grinder was obtained from Zhejiang Meibi Experiment Equipment Co., Ltd. (Zhejiang, China). Microporous membrane filters (0.22 µm) were purchased from Tianjin Jinteng Experiment Equipment Co., Ltd. (Tianjin, China).
